# Imageless robotic-assisted revision arthroplasty from UKA to TKA: Surgical technique and case-control study compared with primary robotic TKA

**DOI:** 10.1007/s00132-021-04182-w

**Published:** 2021-10-29

**Authors:** Lars-Rene Tuecking, Peter Savov, Henning Windhagen, Simon Jennings, Dinesh Nathwani, Max Ettinger

**Affiliations:** 1 Department of Orthopaedic Surgery, Hannover Medical School, Diakovere Annastift, Anna von Borries Str. 1–6, 30625 Hannover, Germany; 2 London North West University Healthcare NHS Trust, Acton Lane Park Royal, London, UK; 3 Charing Cross Hospital, Imperial College London, Fulham Palace Road, London, UK

**Keywords:** Retrospective study, Case-control study, Accuracy, Revision arthroplasty, Radiographic analysis, Retrospektive Studie, Fall-Kontroll-Studie, Präzision, Revisionsendoprothetik, Röntgenuntersuchung

## Abstract

**Background and objective:**

It is evident from the national joint registries that numbers of revision knee arthroplasty operations are rising. The aim of this article is to introduce a new robotic-assisted approach in UKA to TKA revision arthroplasty and investigate the alignment accuracy, implant component use and surgery time and to compare it to primary robotic-assisted TKA arthroplasty.

**Methods:**

This retrospective, case-control study included patients undergoing image-less robotic-assisted revision arthroplasty from UKA to TKA (*n* = 20) and patients undergoing image-less robotic-assisted primary TKA (control group, *n* = 20) from 11/2018 to 07/2020. The control group was matched based on the BMI and natural alignment. Comparison of groups was based on postoperative alignment, outlier rate, tibial insert size, lateral bone resection depth, incision-to-wound closure time. All surgeries were performed by a single senior surgeon using the same bi-cruciate stabilizing TKA system. Statistical analysis consisted of parametric t‑testing and Fisher’s exact test with a level of significance of *p* < 0.05.

**Results:**

The two groups showed no differences in mean BMI, natural alignment (*p* > 0.05) and mean overall limb alignment. No outlier was found for OLA and slope analysis. The smallest insert size (9 mm) was used in 70% of the cases in the revision group (*n* = 14) and in 90% of the cases in the primary group (*n* = 18, *p* = 0.24), distal femoral and tibial resection depth showed no statistical difference (*p* > 0.05). The incision to wound closure time was longer in the revision group but showed no significant difference.

**Conclusion:**

Image-less robotic-assisted revision arthroplasty from UKA to TKA showed a comparable surgery time, and alignment accuracy in comparison to primary robotic-assisted TKA. Comparable bone preservation and subsequent tibial insert size use was observed for both groups.

## Abstract (de)

**Hintergrund und Ziel:**

Die Zahl der notwendigen Revisionsoperationen für Knietotalendoprothesen (TEP) steigt zunehmend. Ziel dieser Studie ist es, eine neue roboterassistierte Technik in der Revision von Schlitten- (UKA) auf Totalendoprothese (TEP) vorzustellen und die Ausrichtungsgenauigkeit, die Verwendung von Implantatkomponenten sowie die Operationszeit zu untersuchen und mit der primären roboterassistierten Endoprothetik zu vergleichen.

**Methodik:**

Diese retrospektive Fall-Kontroll-Studie schloss Patienten ein, die sich im Zeitraum von 11/2018 bis 07/2020 einer bildlosen roboterassistierten Revisionsoperation von UKA zu TKA (*n* = 20) unterzogen, sowie Patienten, bei denen eine bildlose roboterassistierte primäre TEP-Operation durchgeführt wurde (Kontrollgruppe, *n* = 20). Die Kontrollgruppe wurde auf Basis des BMI und der natürlichen Beinanatomie gematcht. Der Vergleich der Gruppen basierte auf der postoperativen Komponentenausrichtung, der Ausreißerquote, der Größe des Onlays, der Tiefe der lateralen Knochenresektion und der Schnittnahtzeit. Alle Operationen wurden von einem einzigen Chirurgen unter Verwendung desselben TEP-Systems durchgeführt. Die statistische Auswertung bestand aus einem parametrischen t‑Test und einem Fischer-Exakt-Test mit einem Signifikanzniveau von *p* < 0,05.

**Ergebnisse:**

Die Gruppen zeigten keinen Unterschied des BMI, der natürlichen Beinanatomie (*p* > 0,05) und der postoperativen Ganzbeinachse. Die Ausreißerquote war in beiden Gruppen für die koronare und sagittale Komponentenausrichtung vergleichbar. Für die Ganzbeinachse und in der Slope-Analyse wurde in beiden Gruppen kein Ausreißer gefunden. Die kleinste Onlaygröße (9 mm) wurde in 70 % der Fälle in der Revisionsgruppe (*n* = 14) und in 90 % der Fälle in der Primärgruppe (*n* = 18, *p* = 0,24) verwendet. Die distale femorale und tibiale Resektionstiefe zeigte keinen statistischen Unterschied (*p* > 0,05). Die Schnittnahtzeit war in der Revisionsgruppe länger, zeigte aber keinen signifikanten Unterschied.

**Schlussfolgerung:**

Die bildlose roboterassistierte Revisionsendoprothetik von UKA zu TKA zeigt eine vergleichbare Operationszeit und Ausrichtungsgenauigkeit im Vergleich zur primären roboterassistierten Knietotalendoprothetik. Für beide Gruppen wurden eine vergleichbare Knochenresektion und konsekutiv vergleichbare Onlaygrößen beobachtet.

Due to increasing numbers of primary implantations of knee endoprostheses, there are also an increasing number of necessary revision procedures. Simultaneously, the number of necessary conversions from UKA to TKA are also increasing. Furthermore, previous clinical outcomes have been shown to be suboptimal after conversion from UKA to TKA compared to primary TKA [4, 5, 11, 13, 27]. In this study, a robotic-assisted technique for conversion of UKA to TKA is presented and the accuracy of this technique is compared. The aim is to improve the accuracy of the outcome and to achieve an improvement of the clinical results in the medium term.

## Introduction

Unicompartmental knee arthroplasty (UKA) is an established and successful procedure in contemporary orthopaedic surgery. In addition to the benefits of UKA implantation, such as bone conserving, ligament-sparing procedure, and restoration of normal knee kinematics, the UKA procedure continues to show higher revision rates in national prosthesis registries in comparison to total knee arthroplasty (TKA) procedures [8, 14, 15, 20]. The UKA procedure is technically demanding and depends mainly on the experience of the surgeon [17]. The British National Joint Registry (NJR) showed that the revision rate for UKA is significantly higher for surgeons with < 30 UKA procedures per year (4% vs. 1% annual revision rate) [16]. In recent years, higher numbers of UKA procedures have been observed in the NJR for arthroplasty in Germany (EPRD, Endoprothesenregister Deutschland). The number of UKA procedures of all primary knee arthroplasty procedures in 2019 was 13.5% and about 4% higher than the previous value in 2015 [6]. For this reason, with an increasing number of UKA procedures, an increase in the number of necessary UKA to TKA revisions can be expected in future years. Furthermore, conflicting evidence still exists regarding clinical outcome and revision rates after UKA to TKA procedures, especially when compared with primary TKA procedures [4, 5, 11, 13, 17, 21, 27]. Isolated case series and case-control studies have demonstrated comparable clinical outcomes and revision rates for UKA to TKA procedures compared with primary TKA procedures [17, 21]. In contrast, recent registry studies from Sweden and Norway and further meta-analyses showed reduced clinical outcomes and higher re-revision rates for UKA to TKA procedures compared to primary TKA [4, 5, 11, 13, 27]. Some authors suspected the reason for this is the mostly necessary use of augmentations, stems and higher inlays due to the increased bone loss compared to primary TKA [27]. In most cases, the increased bone loss during these revision surgeries is due to bone loss during implant removal and resection. As a result, larger inserts and more constrained inserts often have to be used in UKA to TKA revisions [22]. Robotic-assisted primary TKA (RA-TKA) is increasingly gaining popularity in the orthopaedic domain [9]. The main advantages of RA-TKA include improved component alignment and real-time control of soft tissue balance [12, 19, 23, 26, 31]. The use of RA-TKA in the context of UKA revision is a promising approach to improve the results of these revision procedures. The use of robotic assistance might reduce alignment outlier, intraoperative bone loss, and improve soft tissue balance. For this reason, the aim of this study was to examine the extent to which alignment goals can be achieved and bone-saving surgery can be performed. In addition, the workflow of imageless robotic-assisted UKA revision is presented.

## Methods

### Study design

This retrospective case control study consisted of two groups including a total of 40 patients. The first 20 consecutive cases of robotic-assisted UKA revision procedures from November 2018 to July 2020 were included in the experimental group. A matched control group consisted of 20 patients undergoing primary robotic TKA surgery in the same time interval matched by body mass index (BMI ± 5 kg/m^2^) and natural overall limb alignment (nOLA ± 5°). All surgeries were performed by a single senior surgeon (ME) using a bi-cruciate stabilizing TKA system (Journey II ® BCS, Smith & Nephew, London, UK) and an imageless robotic system (NAVIO®, Smith & Nephew) functionally aligned with respect to the preoperative soft tissue balance and prearthritic anatomy. Cases were analyzed for mode of failure (only UKA cases), used implant and augmentation components, insert type, bi-cruciate stabilized insert (BCS) or condylar constrained insert type (CCK), both are available for the Journey II ® BCS prosthesis, postoperative alignment, insert size, lateral bone resection depth, incision to wound closure time (ICT). Radiological analysis consisted of postoperative coronal and sagittal alignment, overall limb alignment (OLA), medial proximal tibia angle (mPTA), lateral distal femoral angle (lDFA) and tibial slope. Radiological analysis consisted of full leg radiographs and true lateral long radiographs. Preoperative natural alignment was either analyzed by full leg radiographs of the contralateral side or by radiographs available prior to primary UKA surgery within the revision group. Standard full leg radiographs prior to primary TKA surgery were used for group matching in the control group. Outliers were defined as valgus overall limb alignment (OLA), severe tibial varus alignment with mPTA < 87° or excessive varus overall limb alignment < 175°, and negative slope or > 6° (aiming for 3°). Statistical evaluation was performed using Microsoft Excel 2019 and GraphPad Prism 9 software (GraphPad Software, San Diego, CA, USA). Numeric parameters are described as mean values and standard deviation (±SD). Group comparisons were done using unpaired t‑tests and Fisher’s exact test. The level of statistical significance was set at *p* ≤ 0.05.

### Surgical technique

Robotic-assisted UKA conversion to TKA and primary RA-TKA were both done with the image-less robotic system NAVIO^TM^ (Smith & Nephew, London, UK) using the Journey II BCS prothesis. Primary RA-TKA was performed as described before [7, 23]. In the following, the technique of robotic-assisted UKA conversion to TKA is described:

Due to the imageless system no preoperative CT scan is necessary. Preoperative analysis included full leg radiographs to determine OLA, mPTA and lateral distal femoral angle (lDFA). Additionally, preoperative full leg radiographs prior to UKA implantation were analyzed to determine the natural alignment and joint line height of the patient. If no radiographs prior to UKA implantation existed, full leg radiographs of the contralateral lesser extremity were used to determine the natural alignment. The intraoperative set-up was comparable to the already published set-up [1, 7].

#### Step 1: Incision, array positioning, registration and soft tissue balance tracking

**Fig. 1:**
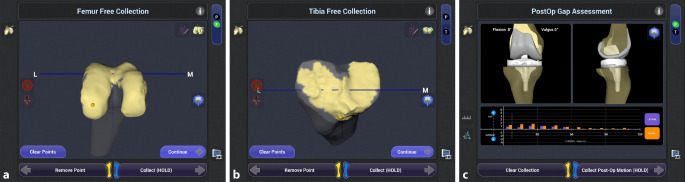
Intraoperative setting with positioning of the components and postoperative evaluation of soft tissue balance. Intraoperative illustration of anatomy mapping of the femur (**a**) and the tibia (**b**) with medial UKA in situ, which is best seen in **a** with smooth surface in the anatomy mapping screen (**a**, **b**). Postoperative gap assessment of medial and lateral laxity, showing medial (*orange*) and lateral (*purple*) gaps within targeted boundaries (**c**)

A standard medial parapatellar approach is used, while the proximal incision is 1–2 cm longer to enable pin position on the femur within the same incision. Tibial pins are attached anteromedially 8–10 cm distal to the tibial plateau to avoid conflicts with the saw. All pins are attached within the bone bicortically. Definition of the hip/knee center and range of motion (ROM) is done by the developer’s standard following the workflow of the NAVIO^TM^ software (Fig. 1). Bone tracking with the handpiece and tracking of the soft tissue balance is done with UKA implants in situ. To achieve adequate varus and valgus stress in soft tissue balance, examination spacer blocks or Hohmann hooks can be used. Assessment of the soft tissue balance in 0° and 90° and within the range of flexion from 0° to 90° should be tracked carefully to enable perfect soft tissue balance in component positioning.

#### Step 2: Planning of component alignment

The aim of any arthroplasty surgery is to reconstruct the joint line and joint line obliquity with respect to the posterior condylar axis. Bone resection depth should not exceed 5.5–6.5 mm lateral (distal + posterior) and, if possible, 7.5–8.5 mm medial (distal + posterior, note the bone loss during implant removal and the thickness of the implant while assessing the medial cut depth) to avoid joint line proximalization. Alignment of the femoral component is done using the 3D planning intraoperative software to verify reconstruction of the posterior condylar axis and the posterior condylar off-set. The femoral component alignment is adjusted to the preoperative detected natural limb alignment (particularly lDFA). Gaps are then balanced especially in 0° and 90° flexion with symmetrical gaps of 1–2 mm mediolaterally. A slight lateral lift-off in flexion is accepted. Adaption of component alignment to gain optimal gap balancing is mainly done virtually prior to execution of cuts. Exceeding limits of 87° of mPTA are avoided An mPTA in excess of 87° should be avoided.

#### Step 3: Implant removal and bone cutting

After virtual alignment of the prothesis components the UKA implants are removed. The distal cut of the femur is done with the trephine following the intraoperative plan determined preoperatively with the NAVIO^TM^ software. Later, 4‑in‑1 cutting blocks are used to perform the last femoral cuts with navigated cutting blocks. The tibial side can be burred alone or preparing by a bone saw with tibial cutting blocks. A probe can be inserted to check gap balancing and postoperative alignment with the NAVIO^TM^ system. If balance differences or limitation of full ROM exist, further bone cuts or soft tissue balancing might be done. Bone defects above 5 mm are compensated with appropriate augmentation.

#### Step 4: Component implantation

After correct execution of bone cuts, component implantation can be done following the clinical routine. After cementing a probe inlay (BCS vs. CCK) and different PE thicknesses can be tested to achieve best soft tissue balance and stability.

## Results

**Fig. 2:**
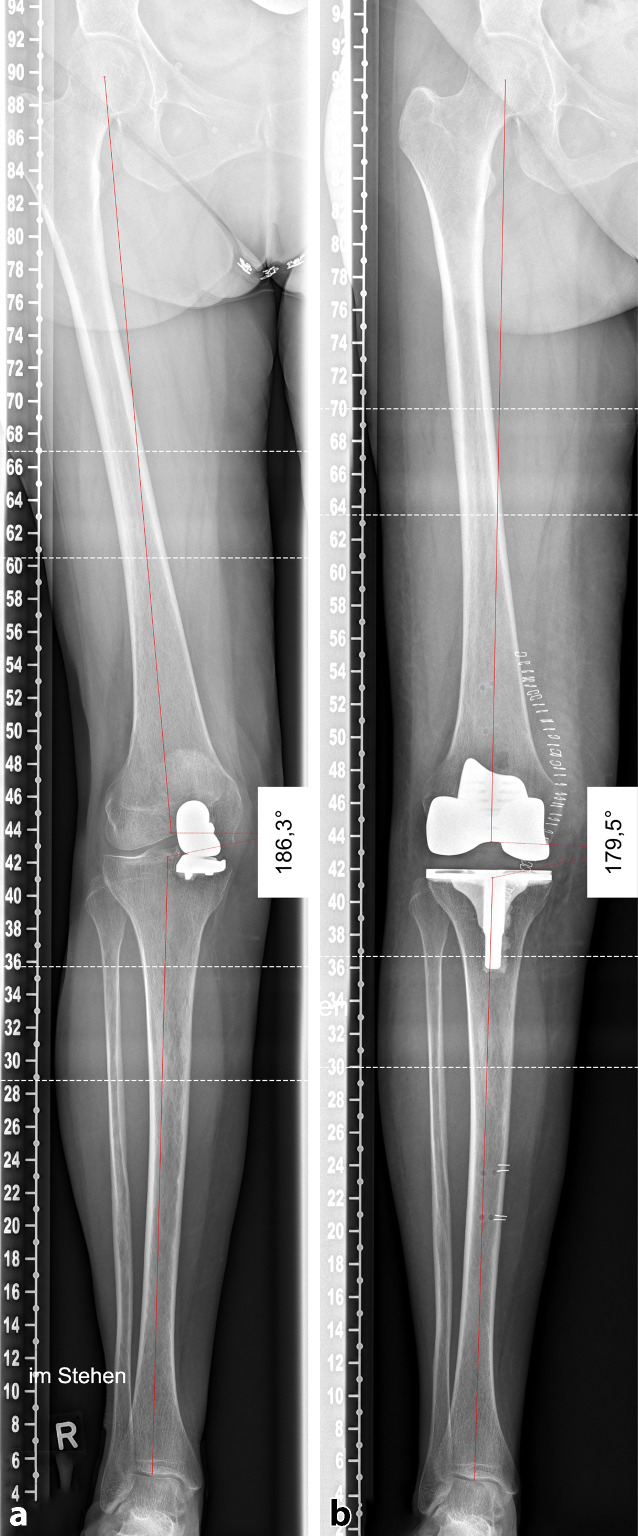
Preoperative and postoperative alignment after UKA to TKA conversion. Preoperative alignment after UKA implantation (**a**) shows a valgus overstuffing (6°) with chronic MCL pain. Postoperative alignment (**b**) after robotic-assisted conversion from UKA to TKA with neutral overall limb alignment

Demographic data and alignment analysis results are shown in detail in Table 1. Both groups showed no difference of mean BMI (*p* = 0.0149) or preoperative natural coronal limb alignment (*p* = 0.151). Reasons of UKA revision were aseptic loosening (*n* = 8, 40%), secondary instability (*n* = 5, 25%), osteoarthritis progression (*n* = 4, 20%) and valgus overstuffing (*n* = 3, 15%). Preoperative mPTA and slope showed no differences between groups (*p* = 0.627; *p* = 0.941 respectively), whereas preoperative lDFA showed more valgus alignment in the UKA revision group (85.5° vs. 87.8°, *p* = 0.03). Incision to wound closure time was higher in the UKA revision group (76.0 min vs. 69.6 min) but did not reach significance level (*p* = 0.052). Postoperative overall limb alignment showed a slightly more varus limb alignment in the primary TKA group (178.6° vs. 176.0°), whereas all postoperative alignment parameters showed no statistical differences between the groups (Table 1). Coronal and sagittal alignment accuracy was found to be similar between groups with no outliers for OLA and slope analysis in both groups (Fig. 2, Table 2) and the same outlier rate for mPTA analysis (5% each, *n* = 1). Lateral bone cut depth on the tibial and femoral side was comparable in both groups (Table 1). Standard BCS inserts were used in 100% of the cases within the primary TKA group and in 90% (*n* = 18) of the UKA conversion cases (*p* = 0.49). Hence, CCK inserts were used in two cases of the UKA revision group (10%). No metal augmentation (stems, femoral augments) was used in any of the cases. Mean insert size was slightly higher in the UKA revision group but showed no statistical difference (9.6 mm vs. 9.1 mm, *p* = 0.11). The minimum onlay size was used in most of the cases in the UKA revision group (70%) but less frequently when compared to the primary TKA group (90%, *p* = 0.24).

**Table 1:** Demographic and results data of both groups

		Robotic UKA revision		Robotic primary TKA		*P* value
Group size (*n*)		20		20		–
Age (years)		62.4	±10.2	68.9	±9.25	*
						0.046
BMI		31.7	±6.8	28.8	±6.6	n. s.
						0.149
ICT (min)		76.0	±11.2	69.6	±16.1	n. s.
						0.052
OLA (°)	Preoperative	177.1	±3.0	175.2	±2.7	n. s.
						0.151
	Postoperative	178.6	±1.9	176.0	±2.5	n. s.
						0.221
mPTA (°)	Preoperative	86.5	±2.5	86.0	±2.0	n. s.
						0.627
	Postoperative	88.5	±1.5	88.9	±1.1	n. s.
						0.837
lDFA (°)	Preoperative	85.5	±3.2	87.8	±1.7	*
						0.030
	Postoperative	87.6	±2.2	89.5	±2.5	n. s.
						0.493
Slope (°)	Preoperative	4.9	±3.4	4.6	±2.9	n. s.
						0.941
	Postoperative	2.3	±0.6	2.6	±1.7	n. s.
						0.857
Lateral cut depth (femoral, mm)	Distal	6.5	±2.0	7.1	±1.7	n. s.
						0.437
	Posterior	7.8	±0.7	7.5	±1.3	n. s.
						0.478
Lateral cut depth (tibial, mm)		10.0	±1.6	10.0	±1.1	n. s.
						0.941
Onlay size (mm)		9.6	±1.1	9.1	±0.3	n. s.
						0.112
	*n* of minimal size (% of total)	14	(70%)	18	(90%)	n. s.
						0.240

Values in mean ± SD

*SD* standard deviation, *UKA* unicompartmental knee arthroplasty, *TKA* total knee arthroplasty, *ICT* incision to wound-closure time, *BMI* body mass index, *OLA* overall limb alignment, *mPTA* medial proximal tibia angle, *lDFA* lateral distal femoral angle, *Lat.* lateral, * significance level, *n.s. *not significant

**Table 2:** Outlier rate of postoperative alignment

		Robotic UKA revision		Robotic primary TKA		*P* value
Group size (*n*)		20		20		–
OLA outlier rate	*n* outlier	0	(0%)	0	(0%)	n. s.
	(% of total)					–
mPTA outlier rate	*n* outlier	1	(5%)	1	(5%)	n. s.
	(% of total)					> 0.999
Slope outlier rate	*n* outlier	0	(0%)	0	(0%)	n. s.
	(% of total)					–

Values in mean ± SD

*SD* standard deviation, *UKA* unicompartmental knee arthroplasty, *TKA* total knee arthroplasty, *OLA* overall limb alignment, *mPTA* medial proximal tibial angle, *n.s*. not significant

## Discussion

The main result of this retrospective case control study is that robotic-assisted conversion from UKA to TKA is a precise technique in revision arthroplasty and shows similar alignment outcome parameters when compared to primary RA-TKA.

Alignment accuracy of imageless robotic-assisted UKA conversion to TKA was similar to primary RA-TKA. No radiological outliers for OLA and slope analysis were found in either of the groups. Furthermore, postoperative coronal alignment parameters were all comparable between the groups. In addition to the numerous published studies on improved alignment accuracy in primary robotic-assisted knee arthroplasty in comparison to conventional TKA [12, 19, 23, 26, 31], this case series was able to demonstrate a comparable accuracy and outcome to robotic-assisted primary knee arthroplasty. Intraoperative control of component alignment and joint line restoration in revision arthroplasty is often a challenge due to further bone loss or loss of bony reference points after implant removal. Therefore, malalignment after manual UKA conversion to TKA might be reduced with this robotic-assisted approach. Several studies reported a decreased clinical outcome after UKA to TKA revision when compared to primary TKA. These studies showed an increased use of metal augmentation, higher constraint level and increase of polyethylene (PE) thickness in UKA to TKA revision groups [11, 22, 29]. Contrary to the published data, a comparable PE thickness was observed in the current case series for UKA revision in comparison to the primary group and the minimum PE size could be used in the majority of the UKA revision cases (70%). Additionally, the robotic-assisted approach enables the intraoperative control of soft tissue balance, which might support the use of standard primary implants and minimum size PE onlays instead of constrained implants and onlays. In this context, image-based robotic-assisted UKA revision also showed comparable and a tendency towards lower PE thickness compared to manual UKA revision in a recently published study [30]. Alignment parameters were not analyzed in that study. Thus, no comparison between imageless and image-based robotic-assisted UKA revision is possible at this point.

Furthermore, in the UKA revision cases in this series, the use of augments or stems was not required. Individual studies report the need for augments or stems in 30–54% of UKA to TKA revisions [2, 29]. Additionally, considering the lateral bone resection height in our study, which was comparable between both groups on the tibial and femoral side, it can be assumed that a bone-conserving revision of UKA to TKA is possible by robotic assistance. Nevertheless, two cases of the UKA revision group (10%) needed a semiconstrained insert, while none of the primary TKA cases needed a CCK insert. Comparable studies by Sarraf et al. [22] and Lunebourg et al. [18] reported the use of constrained implants in 4.2–10.4% of UKA to TKA revision cases. Nevertheless, the exact level of constraint remains unclear.

Despite the technically more difficult procedure of UKA revision, the time from incision to closure was only slightly higher in the UKA revision group compared to the primary TKA group (76.0 min vs. 69.6 min), this difference also did not reach significance level. The incision to wound closure times (ICT) determined in this study are to be compared to frequently published data of single center studies or registry studies [3, 10, 24, 25, 28]. Published mean ICT range from 67 min to 104 min, regardless of whether manual or robotic-assisted TKA was used [3, 10, 24, 25, 28]. Therefore, robotic-assisted UKA conversion is comparable to primary arthroplasty in terms of average time required and does not lead to a significant increase in time from incision to closure.

This study has several limitations. First of all, this was a retrospective study of the first 20 consecutive cases of robotic-assisted revision from UKA to TKA analyzing objective operative parameters and alignment outcome only. Thus, the statistical power is reduced. Moreover, the control group was matched by BMI and natural alignment. In some UKA revision cases, preoperative full leg radiographs prior to UKA implantation were not available. Thus, a full leg radiograph of the contralateral side, as performed in the clinical routine, was used to identify the natural alignment. Nevertheless, natural alignment of both lower extremities might differ significantly and therefore might have influenced the classification. Furthermore, no clinical outcome data were obtained because this was not the subject to this study; however, this would give further important information about robotic-assisted UKA revision and should be investigated in further studies. In addition, this study compared UKA revision with primary robotic arthroplasty. No comparison was made between manual and robotic-assisted UKA revision. The aim of this study was to verify whether robotic-assisted UKA revision is comparable to primary arthroplasty in the parameters investigated. The aim was to improve the surgical technique to bring the results of UKA revision in line with primary arthroplasty. Nevertheless, a manual UKA revision comparison group would help to determine whether the robotic-assisted technique could achieve an improvement in the accuracy of this operation in a direct comparison.

## Conclusion

- Robotic-assisted revision from UKA to TKA is a reliable approach with accurate component alignment.
- Robotic-assisted revision from UKA to TKA might help to preserve bone stock and might avoid using revision augmentation material and higher constraint implants.
- Surgery time of robotic-assisted revision from UKA to TKA is comparable to primary TKA.
- Evidence whether these improvements have an impact on clinical outcome is pending and should be further investigated.

## Abbreviations

BCS: Bi-cruciate stabilized insert
BMI: Body mass index
CCK: Condylar constrained insert type
EPRD: Endoprosthesis Register Germany
ICT: Incision to wound closure time
lDFA: Lateral distal femoral angle
MCL: Medial collateral ligament
mPTA: Medial proximal tibial alignment
NJR: National Joint Register
OLA: Overall limb alignment
PE: Polyethylene
ROM: Range of movement
TKA: Total knee arthroplasty
UKA: Unicompartmental knee arthroplasty
